# Almost nothing is known about the tiger shark in South Atlantic waters

**DOI:** 10.7717/peerj.14750

**Published:** 2023-01-20

**Authors:** Samuel Balanin, Rachel Ann Hauser-Davis, Eloísa Giareta, Patricia Charvet, Natascha Wosnick

**Affiliations:** 1 Projeto Tintureira—Associação MarBrasil, Pontal do Paraná, Brazil; 2 Programa de Pós-graduação em Zoologia—Universidade Federal do Paraná, Curitiba, Paraná; 3 Laboratório de Avaliação e Promoção a Saúde Ambiental, Instituto Oswaldo Cruz, Oswaldo Cruz Foundation, Rio de Janeiro, Brazil; 4 Programa de Pós-graduação em Sistemática, Uso e Conservação da Biodiversidade—Universidade Federal do Ceará, Fortaleza, Brazil

**Keywords:** Conservation efforts, Geographical distribution, Elasmobranch, Population genetics, Fisheries bycatch, Management, Feeding ecology, Human interactions, Movements and migration

## Abstract

The tiger shark (*Galeocerdo cuvier*) has been relatively well assessed concerning biology and ecology aspects in both Atlantic and Pacific North America and in Caribbean waters. The amount of data in these regions has led to the species protection under capture quotas and with the creation of sanctuaries. The reality in developing countries, however, is the exact opposite, with scarce information on the species in the southern hemisphere, namely South American and African waters. In these regions, protection measures are insufficient, and studies on tiger shark biology and ecology are scarce, significantly hindering conservation and management efforts. Thus, the aim of this study was to compile scientific literature on the tiger shark in the South Atlantic and discuss the impact of these data (or lack thereof) distributed within a total of ten research categories for guiding management plans. In total, 41 scientific publications on different *G. cuvier* biology and ecology aspects were obtained. The most studied topics were Feeding Ecology (*n* = 12), followed by Human Interactions (*n* = 8), and Movements and Migration (*n* = 7). Northeastern Brazil (Southwest Atlantic) was the most researched area, probably due to the higher coastal abundance of tiger sharks in this area, alongside a high number of recorded attacks, justifying funding for studies in the region. No studies carried out in other South American or African countries were found. It is important to mention that even though some research topics are relatively well covered, a severe knowledge gap is noted for risk assessments and fisheries management, with a proposition for the implementation of sanctuaries noted. This is, however, particularly worrisome, as the South Atlantic is mostly unexplored in this regard for tiger sharks. It is also important to note how different the attention given to this species is in the North Atlantic when compared to the South region. Lastly, we highlight that the existence of sub-populations, the lack of migratory corridors geographically connecting distinct areas used by the species, and the lack of fisheries statistics on tiger shark landings, all increase the vulnerability of this species in the South Atlantic.

## Introduction

The tiger shark, *Galeocerdo cuvier*, is the largest Carcharhinidae member and one of the largest apex marine predators (Meyer et al., 2014). It is also one of the most recognizable sharks worldwide, displaying a circumglobal distribution in both tropical and temperate waters. It is widely known for its opportunistic feeding habits, which include both normal shark prey and a wide variety of unusual items (Simpfendorfer, Goodreid & McAuley, 2001; Dicken et al., 2017). Like many elasmobranchs, tiger sharks comprise an important ecosystemic resource, structuring marine environments, maintaining food web stability and nutrient and energy cycling between trophic levels, as well as contributing to prey population control (Aguiar & Valentin, 2010; Estes et al., 2016; Motivarash Yagnesh et al., 2020). The tiger shark, however, as many other elasmobranch species, is highly at risk due to several negative marine environment pressures. It is currently listed as Near Threatened at a global level based on declines of about 30% in the past three generations (53–68 years), mostly due to fisheries exploitation and mortality resulting from shark control programs (Roff et al., 2018), displaying a decreasing population trend with evidence of fragmented populations (Ferreira & Simpfendorfer, 2019).

A recent review published by Holland et al. (2019) has reported several knowledge gaps regarding tiger sharks, indicating priority fields for future research concerning this species. It is interesting to note, however, that tiger sharks have consistently received greater attention in the North Atlantic when compared to the South region. For example, basic tiger shark biology data has been available for the North Atlantic since 1949 (Gudger, 1949), and several Northern hemisphere regions have made use of such a high available data volume to direct conservation actions (Driggers et al., 2008; Carlson et al., 2012; Hammerschlag et al., 2012; Graham et al., 2016; Sulikowski et al., 2016; Hammerschlag et al., 2017; Morgan et al., 2020). Underdeveloped and developing countries, however, have not matched these research efforts, due to a suite of issues, ranging from scarce or non-existent fishing regulations and fisheries statistics, lack of financial support, government funding cuts, and the still prohibitively high costs of several research methods and technologies in these regions, hindering shark fisheries management (Castello et al., 2009; Zerbe, 2013; Wade, 2016; MMA, 2014; Barreto et al., 2017; Reis-Filho & Leduc, 2017; Booth, Squires & Milner-Gulland, 2019; FAO, 2020; McManus & Neves, 2021). Thus, tiger shark biology and ecology assessments in these areas are still significantly lacking on several fronts, significantly hindering conservation and management efforts.

In this regard, to better understand research and conservation priorities, knowledge gaps, and future research needs in the South Atlantic, this study aimed to compile literature data on this species in this region, also discussing the impact of these data (or lack thereof) in directing risk assessments and guiding management plans. We also highlight research topics requiring further investigation, as well as emerging research opportunities, which may aid in directing research groups, funding agencies, and policymakers.

## Survey Methodology

**Figure 1:**
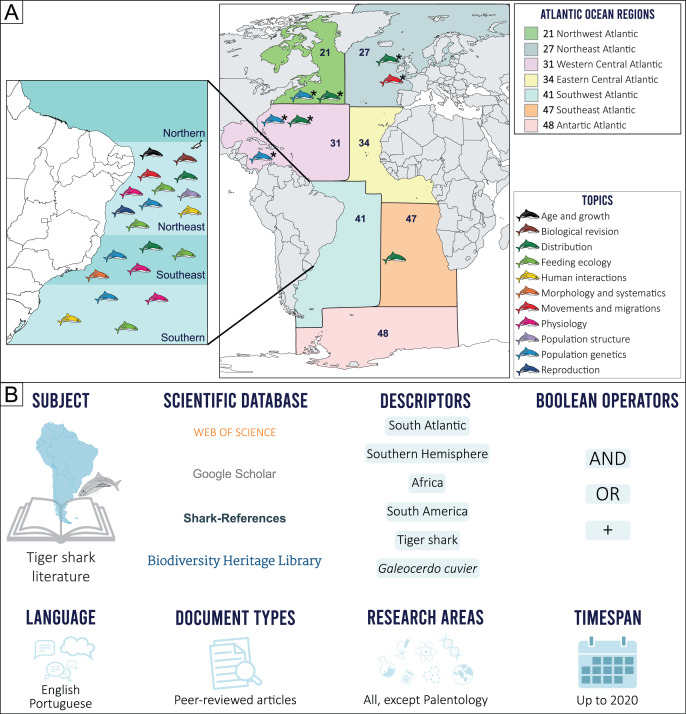
(A) Topic distribution discussed in this review throughout the Atlantic Ocean. (B) Scienciometric search method. *Refers to studies performed in other regions that present data on South Atlantic tiger shark populations.

The geographical limits considered in the present study were established prior to our scientometric search, by determining the border between the North and South Atlantic oceans, based on the Equator counter-current, with the South Atlantic delimited by the Pacific Ocean on the west, the Indian Ocean on the east and the Arctic Sea on the south (International Hydrographic Organization, 1953). This delimitation, however, excludes most of northern Brazil (*i.e*., the state of Macapá), an area more associated with the South Atlantic Ocean due to characteristic ecological and fisheries features. Because of this, the Food and Agriculture Organization (FAO) Fishing Major Areas (FAO, 2022) were employed as reference, as these areas include all the geographic coordinates of the 41 (Brazil, Uruguay, and Argentina) and 47 (Gabon, Congo, Democratic Republic of Congo, Angola, Namibia, Botswana, and South Africa) regions that comprise the Southwestern and Southeastern Atlantic, respectively (Fig. 1A).

Tiger shark studies in scientific publications were searched for in indexed databases. A structured Boolean search (AND, OR, +) was performed using the following search terms: “South Atlantic”, “Southern Hemisphere”, “Africa”, “South America”, “tiger shark”, and “*Galeocerdo cuvier*”. A snowball method was then applied to search for additional publications from the reference lists. Only peer-reviewed publications were included. Documents in English and Portuguese up to 2020 were considered (Fig. 1B). After a manual screening of titles and abstracts and excluding duplicates and articles that did not report tiger shark data, a total of 41 records were selected and included in the final analyses. Research articles that did not necessarily focus on the species but that somehow contributed with relevant information were also included (*e.g*., commercial fisheries landing composition, traditional ecological knowledge, regional elasmobranch checklists). The retrieved studies were then categorized according to major and minor themes established by the authors (Table S1), and classified into ten major topics, as follows: Morphology and Systematics, Reproduction, Age and Growth, Feeding Ecology, Physiology, Population Structure, Human Interactions, Movements and Migration, Distribution, and Population Genetics. The Human Interactions topic includes both Fisheries and Attacks subtopics, and the Feedings topic also includes information on Local Fisher Knowledge (LFK) studies that contribute meaningfully to our research aims. No specific studies on tiger shark Conservation and Management were found, so this theme is discussed alongside other topics with relevant information on the subject.

## Results

**Figure 2:**
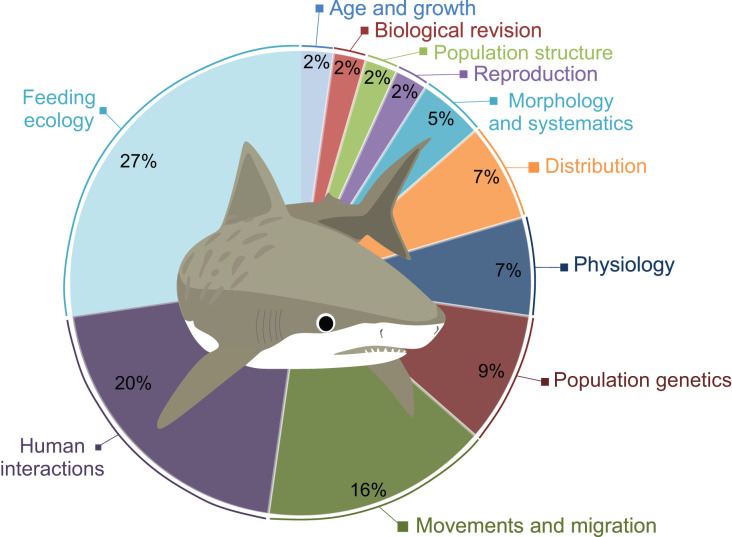
Percentage of publications retrieved in this review per major topic.

A total of 41 scientific publications on different *G. cuvier* biology and ecology aspects were obtained in our search, presented in Table S1. The topics most studied was Feeding Ecology (*n* = 12), followed by Human Interactions (*n* = 8), and Movements and Migration (*n* = 7). A total of four studies on Populations Genetics and three on Physiology were also retrieved. Morphology and Systematics, and Population Structure are covered in two publications each. Lastly, only one paper on Reproduction, one on Age and Growth, and one on Distribution were found (Fig. 2).

**Figure 3:**
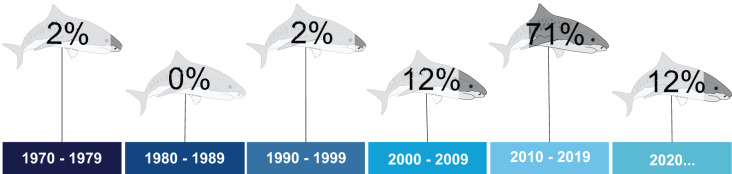
Percentage of publications on tiger sharks throughout the assessed decades.

The first study dates from 1977, and was conducted in the state of Ceará, Northeastern Brazil (Alves, 1977). For two decades, this remained the only article published for tiger sharks in the South Atlantic. No articles were published in the 1980s (Fig. 3). The temporal distribution indicates a peak in studies between 2010 and 2019 (71%). More recently, although the 2020 decade has barely begun, an increasing number of publications was noted (Fig. 3). Concerning temporality for other countries, no studies carried out in South American or African countries are available.

Most assessments were carried out in Northeastern Brazil (Southwest Atlantic) (*n* = 22) (Fig. 3), probably due to the higher coastal abundance of tiger sharks in this area, alongside a high number of attacks recorded in the city of Recife, in the state of Pernambuco. Moreover, a well-known tiger shark incursion hotspot is noted at the Fernando de Noronha Archipelago (Southwest Atlantic), clearly attracting further attention to the species. The studies carried out in Northeastern Brazil mostly focus on tiger shark spatial ecology (*i.e*., movements and migration) (*n* = 8) and attack patterns and drivers in Recife (*n* = 6). Studies carried out in Southern Brazil, on the other hand, have mainly focused on feeding ecology, trophic niche, and the importance of the species to local food chains (*n* = 6). Only 10 studies were identified for other Brazilian regions, focusing on feeding ecology, morphology and systematics, fishing, distributions, and physiology. No studies were found regarding other South American countries (*i.e*., region 41—Southwest Atlantic). Finally, no studies focusing on tiger sharks were identified for the Southeast Atlantic (*i.e*., region 47—African countries).

## Discussion

The discussion is categorized according to each of the subtopics presented in the methodology section.

### Morphology and systematics

Even though the phylogeny of the Carcharhiniformes order based on familial distribution is still debated by specialists, and cases of non-monophyletic groups and subgroups have been reported (Compagno, 1977, 1988), only two studies concerning tiger shark morphology and systematics were obtained in the present review, both carried out in Southeast Brazil.

The first evaluated dermal denticle characteristics in tiger sharks, as this type of assessment comprises a valuable tool in elucidating phylogenetic relationships and is also useful in clarifying evolutionary adaptations (Poscai et al., 2017). The authors characterized the dermal denticles present on the nictitating membrane of the species through scanning electron microscopy. The species specific denticle shape and distribution was hypothesized as associated with a decreased need for eye protection due to tiger shark head shape, although a role in hydrodynamics and ocular protection cannot be discarded. The second study addressed certain phylogenetic aspects by analyzing the caudal muscle morphology of different Carcharhiniform species (de Oliveira, de Carvalho & Soares, 2019). In both studies, tiger shark morphology was more correlated to Sphyrnidae members than to other Carcharhinidae. These data further corroborate the distinct biological and molecular features that tiger sharks display when compared to other Carcharhinidae representatives, a proposed exclusion from Carcharhinidae, and the indication of Sphyrnidae as a sister group proposed by Naylor et al. (2012). This has, in turn, led to the species’ recent reclassification as Galeocerdidae (Ebert, Dando & Fowler, 2021).

The very low number of publications on tiger shark morphology and systematics in the South Atlantic obtained herein clearly indicate the need for further assessments in this regard. However, it is important to emphasize that, apart from some morphological features (*e.g*., reproductive organs, ontogenetic growth), data from other regions can be extrapolated to the South Atlantic, mainly concerning systematics and phylogeny. However, it is also important to note that, even when data was already generated by other more-developed regions, additional studies on South Atlantic waters are still encouraged, not only due to the singular importance of the subject itself, but also as a way to prevent colonizing science issues.

An important step in this direction may comprise taking advantage of dead individuals caught in artisanal and commercial South Atlantic fisheries, following the increasing trend for non-lethal studies on this species noted in other regions (Hammerschlag & Sulikowski, 2011). However, it is important to note that carcass decomposition status should be considered when evaluating dead individuals, and sampling should be standardized to individuals in the same decomposition stage, as what is routinely carried out for other marine megafauna representatives, such as marine mammals, where a standardized carcass decomposition code is employed (Geraci & Lounsbury, 2005). This, on the other hand, is not a limiting factor for certain biological samples (*e.g*., teeth and denticles), of paramount importance for taxonomy and morphology investigations, and opportunistic sampling in these cases is highly encouraged.

### Reproduction

Only one study on reproductive aspects was obtained in our search, conducted in 1977 off the coast of Ceará, a Northeastern Brazilian state in the Western Atlantic (Alves, 1977). That study reported that the presence of spermatozoa in the nidimentary glands of adult females is common, suggesting that spermatozoa can survive for long periods of time in females and that fertilization can occur sometime prior to copulation. The study also indicates that females usually carry no more than 36 embryos, comparatively less than the number of embryos reported for other regions (Alves, 1977). The minimum size for sexual maturation was reported in this first assessment as 237 cm for males and 210 cm for females, with no further details on how the total length was taken (*e.g*., fork length, precaudal length), corroborating previous descriptions by Clark & von Schmidt (1965) for the coast of Florida, in the United States. The authors also report that pregnant females present a gestation period of about 1 year and are found in the area year-round at different pregnancy stages, with more developed embryo stages noted between May and August. Shallower regions (10–25 m) were noted as nursery grounds in the study area. In males, complete reproductive system development and sperm production were reported prior to the complete development of secondary sexual characteristics, such as clasper calcification (Afonso et al., 2012). Neonates (70–90 cm) and mature individuals (>310 cm) were reported only during the first annual quadrant (January–May), suggesting that this period is important for species parturition in Recife, Northern Brazil (Afonso & Hazin, 2014). Although the data presented above cover tiger shark reproductive aspects, it is important to emphasize that this was not the scope of the publications, and because of this, they were not included in the major theme described herein of reproduction. Other assessments not included in our review due to the applied inclusion criteria (only peer-reviewed articles) have further indicated that Southern Brazil has been identified as possible tiger shark parturition and nursery area, given the historical landing of pregnant females in the austral spring, and neonates, and juveniles in the summer and early austral autumn in the state of Paraná (Bornatowski & Abilhoa, 2012). However, no detailed analyses based on nursery area criteria as established by Heupel, Carlson & Simpfendorfer (2007) have been carried out to date, configuring an expressive knowledge gap in this regard.

Reproduction data gaps significantly hinder conservation efforts, as they are paramount in proposing both adequate fisheries management and marine protected areas for specific ontogenetic phases (*i.e*., neonates, juveniles, or pregnant females), especially for nursery areas where neonates and juveniles are more susceptible to human pressure (Heupel et al., 2018; Morgan et al., 2020). We suggest urgent assessments on this topic, and that special attention be given to the southern limits of tiger shark distribution, as different fertility rates and gestational periods may occur due to the lower local temperatures in these regions (Yamaguchi, Taniuchi & Shimizu, 2000; Lombardi-Carlson et al., 2003; Taylor, Harry & Bennett, 2016) requiring differential management plans (Walker, 2005). In addition, as stated in the previous topic, access to dead individuals landed from artisanal and commercial fisheries may also aid in this kind of evaluation, especially as sexing and pregnancy identifications can be easily carried out by non-experts with minimal instruction and do not require special or expensive personnel training. This type of observer-based assessment has been successfully applied in other regions. Furthermore, the use of non-lethal methodologies such as physiological (hormonal profile) and morphological (clasper rigidity and presence of semen in males, presence of mating bite signs in females) parameter evaluations to evaluate reproductive periods, the tagging of mature and pregnant females to identify migratory routes and possible birthing places and the identification of nursery areas, according to Heupel, Carlson & Simpfendorfer (2007) are all suggested to further knowledge in this regard for tiger sharks in the South Atlantic.

### Age and growth

No studies specifically on tiger shark age and growth were found in our search, and only one assessment has been conducted on tiger shark growth rates (but reporting no age estimation) to date in the entire South Atlantic (Afonso et al., 2012). This lone study indicates the highest growth rates described in the literature for the species, as one male individual captured off the coast of Recife, Brazil was sexually mature at 304 cm TL and around 3.5 years, at half the age of global estimates for the species, and one female exhibited a significant growth rate following capture-recapture procedures only a few months apart. The authors indicate that, according to available age-at-length models, both individuals should take at least twice as long to reach their observed dimensions, thus indicating accelerated growth in the Recife area (and, possibly, across the Southwest Atlantic) (Fig. 3), postulated as due to local environmental factors and greater prey availability, although further studies with larger sample sizes are required to elucidate this hypothesis.

As tiger shark growth data (with no age estimation) for the South Atlantic are derived from only one male and one female specimen, extreme caution is necessary if extrapolating these data for practical applications. In addition, no ontogenetic assessments associated with age/growth data are available for the South Atlantic, and studies on how environmental factors such as seasonal variations can affect the growth rates of the species are also lacking. As vertebrae comprise the most efficient way to study shark age and growth, and samples from museum/university collections can still be used for historical descriptions, along with fresh samples from commercial landings, future studies should direct efforts to generate such data for the region. Despite the fact that capture-mark-recapture data require long-term efforts and exhibit low success rates, vertebrae analyses are less-expensive and generate more robust data for risk assessments, for example. Thus, it is imperative that age and growth data be determined for the South Atlantic, so more robust and updated regional risk assessments may be performed. Assessments employing a greater number of tagged animals are also encouraged, as spatial ecology data will benefit not only age and growth knowledge, but also migration and movement patterns that are still completely unknown to tiger sharks outside Northeast Brazil (more specifically, the state of Pernambuco).

### Feeding ecology

A higher number of papers (*n* = 12) was found concerning feeding ecology, separated into dietary habits and trophic ecology for discussion purposes, all conducted in Brazil. The reasons why the amount of information on this topic is predominant in the South Atlantic are uncertain, but may be simply a reflection of greater interest in the topic by research groups.

#### Dietary habits

Concerning dietary habits, several papers retrieved in our review indicate that juvenile tiger sharks captured in Brazilian coastal areas feed mainly on teleosts, other elasmobranchs, birds, and crustaceans (Shibuya, de Souza Rosa & Gadig, 2005; Bornatowski, Robert & Costa, 2007; Bornatowski et al., 2014b), corroborating the species classification as a generalist and opportunistic predator (Bornatowski, Robert & Costa, 2007). Moreover, a preference for highly available prey is noted (Bornatowski, Robert & Costa, 2007, Bornatowski et al., 2014b).

Other important records for the species in Brazil obtained herein also demonstrate opportunistic scavenging habits (Rosas et al., 1992; Di Beneditto, 2004; Bornatowski et al., 2012a, 2012b; Rada et al., 2015), including feeding on other sharks (nurse sharks, *Ginglymostoma cirratum*) in the first report for this type of event worldwide (Rada et al., 2015). This opportunistic scavenging has also been reported in another study in Brazil, although not retrieved in our initial search. That paper reports LFK from fishers from the state of Bahia, Northeastern Brazil, who state that tiger sharks routinely feed on carcasses washed in by floods (Filho & Neto, 2016). Fishers in the same state have also indicated that tiger sharks are strongly associated with river mouths, foraging on potential prey brought by in by currents (Barbosa-Filho et al., 2014) and report that the species usually follow boats to feed on discarded organic waste (Filho & Neto, 2016). Off the coast of Paraíba, Northeastern Brazil, tiger sharks have also been reported as feeding on sea turtles, with most bites found on sea turtle carcasses belonging to this species (Bornatowski et al., 2012a). The presence of recent or healed bite marks on some sea turtle carcasses, however, may indicate both active predation and scavenger habits (Bornatowski et al., 2012a).

In addition to other fish and sea turtles, regional records concerning cetacean and pinniped predation events were also obtained, recorded off the coast of Rio de Janeiro, Southeastern Brazil (Rosas et al., 1992; Di Beneditto, 2004), and a high number of bite marks has been reported in the state of Bahia (Bornatowski et al., 2012b), potentially indicating that tiger sharks also feed on live cetaceans. This is seemingly corroborated by the fact that the tiger shark is reported as following cetaceans during their migrations up the Brazilian coast, from the south towards the Abrolhos Bank, in the Northeast (Bornatowski et al., 2012b; Filho & Neto, 2016). Furthermore, tiger sharks were also observed feeding on dead whales in Abrolhos in another study, responsible for 54% of all bites found on carcasses (Bornatowski et al., 2012b). Interestingly, the first record concerning the predation of a sloth by a tiger shark was obtained for the South Atlantic, in the state of Rio de Janeiro (Miranda & Santos, 2021), although whether the sloth was preyed on when moving between local islands or if the carcass reached the sea and was then consumed *post-mortem* could not be determined.

In Paraná, southern Brazil, tiger sharks have also been reported as feeding on seabirds indicating that a previously described behavior for the Northern hemisphere (Lowe et al., 1996; Gallagher, Jackson & Hammerschlag, 2011) also occurs in the South Atlantic (Bornatowski, Robert & Costa, 2007). This behavior is also noted off the coast of Bahia (Filho & Neto, 2016). However, as these records are not widespread, further investigations are required to understand whether these are punctual foraging events or indeed routine active tiger shark predation habits. Moreover, the level of importance these interactions represent for tiger shark ecology in the region has also not been unraveled to date, and the analyzed individuals comprise mostly juveniles and neonates. Therefore, little is still known about adult and subadult feeding ecology in the South Atlantic, also leading to a complete lack of knowledge on potential ontogenetic dietary shifts. In addition, the studies reported herein focused on animals captured in the Southwest Atlantic. Thus, dietary habit data in oceanic environments and in other South Atlantic regions are also lacking.

#### Trophic ecology

Concerning trophic ecology, data from the state of Paraná indicate that the species occupies a high trophic level in coastal trophic chains, confirming its classification as an apex marine predator (Bornatowski et al., 2014a, 2014b). Thus, declines in species abundance may lead to intense cascade effects, although the ecological consequences of their local extinction are yet to be understood (Bornatowski et al., 2014a). The ecological role and trophic position of the species is well described for other regions (Lowe et al., 1996; Dicken et al., 2017; Ferreira et al., 2015; Aines et al., 2018), thus future research is needed to determine if the same holds true in the South Atlantic. For instance, trophic position data for other South Atlantic regions and trophic interactions between tiger sharks and other species during ontogenetic development are, notably, lacking. Studies on the daily supply and energy requirements used by different phases of ontogenetic development of the species are suggested, as well as aiming to identify ontogenetic and regional variations of the species in the South Atlantic. Food chain analyses, niche overlaps, and position evaluations, as well as studies on potential species removal effects along tiger shark distribution in the South Atlantic, are also paramount.

### Physiology

A total of three studies concerning physiology were obtained in our review. One was carried out at the Fernando de Noronha Archipelago with the aim of investigating capture stress effects and the validation of new stress markers for the species (Wosnick et al., 2017). Data on adults, however, are still lacking, as well as potential ontogenetic stress response variations upon capture. Furthermore, the impacts of both scientific and commercial captures are still not fully understood, and, although evidence of high survival rates for the species upon capture has been reported for the Recife coast (Afonso & Hazin, 2014), the physiological pathways involved in tiger shark recovery following capture are still not fully understood. In another assessment, the potential negative effects of fasting on juvenile tiger sharks were evaluated for the first time in the South Atlantic in the state of Maranhão, in the Fernando de Noronha Archipelago, and in the state of Paraná (Wosnick et al., 2020). According to the authors, the observed condition potentially resulted in serious metabolic consequences by preventing digestion and the recovery of lipid stores, although the causes of this restriction are still unclear and may be associated with other yet unidentified pathologies. In another first-time report, hormone (testosterone, progesterone, and B-estradiol) levels in juvenile male tiger sharks tagged in the Fernando de Noronha Archipelago were assessed (Wosnick et al., 2017). The results indicate that steroid hormones might play an important role in both home-range and migratory shark behavior.

Physiological assessments are paramount in understanding many aspects of shark behavior, migration, and stress responses, among others. In fact, recently physiology has been brought forth as indispensable in addressing current and future anthropogenic stressors that both directly and indirectly affect elasmobranchs (Lyons et al., 2019), as it is clear that physiological impairments may significantly compromise fitness parameters and negatively affect population recruitment (Wosnick et al., 2021). Expansion of comparative studies of hormone levels and migratory behavior, increasing the number of captured and tagged animals, as well as including those that have already reached sexual maturity, are strongly suggested. Data on capture stress from commercial fisheries as well as mortality rates are also necessary, along with stress studies focusing on stress upon capture between life stages and sexes. Lastly, as Southern Brazil encompasses the southern limit of distribution of the species, studies in this region should be prioritized, aiming to elucidate the particularities of this population. Studies aimed at investigating and monitoring the health of tiger sharks are also encouraged, especially considering the ingestion of objects that have the potential to compromise affected individuals.

### Population structure

A single study was obtained regarding tiger shark population structure in our applied geographic range. The single assessment conducted off the coast of Recife indicates that tiger sharks are more abundant between the coastline and the end of the continental shelf, and that reach larger sizes when compared to other shark species that are part of the local assembly (Afonso & Hazin, 2014). The sex ratio was noted as 0.69:1 (M:F) and local distribution was verified as influenced by annual fluctuations directly associated to seasonality. Abundance was also positively correlated with high and low tide amplitudes and low rainfall. The authors report juveniles as the predominant size class in local captures, with few captured animals above the maturity size of 310–320 cm total length (TL) and a high number of neonates (<100 cm TL) in the first annual quadrant, pointing to a parturition period. The abundance of tiger sharks over the 4 years of study was considered low, however, no evidence that this occurs in the long term was found, although factors (*e.g*., temperature change and thermoclines) that might affect tiger shark distribution were not established.

As only a single assessment was obtained concerning population structure, long-term analyses comparing seasonal variation are paramount to better understanding distribution trend shifts, both in Brazil and in other South Atlantic areas. Considering the importance of commercial fisheries in generating shark data in Brazil, landing monitoring to describe tiger shark population structure data is highly recommended. However, such a methodology must be used with caution, as individual patterns of the studied fleets can bias the data, generating under- or overestimated predictions. In this context, telemetry studies as a supplementary methodology are recommended, however, often prohibitive due to the high relative costs of telemetry methods (Lippi et al., 2021), and significant cost differences among different tags (*e.g*., sPAT tags x miniPAT tags—Miller et al., 2019). Moreover, increased sampling periods are suggested, to include a minimum historical series with seasonal variations, as well as regional samplings and comparisons, generating a larger scale database at the East and West Atlantic levels. Abundance associations to abiotic factors such as substrate, turbidity, tide, and temperature, and biotic factors such as prey availability and the presence of competitors, are also required, to understand which factors can influence tiger shark population structure.

### Human interactions

#### Fisheries

Despite being widely distributed worldwide, information on tiger shark landings is still scarce for the South Atlantic. Most of the data in this regard consist of gray literature, not published in peer-reviewed journals. Therefore, only studies that assessed fisheries at a regional level indirectly addressing fishing information were considered herein (six papers). Tiger sharks are occasionally caught as bycatch in coastal artisanal fisheries targeting teleost off the coast of Paraná (Costa & Chaves, 2006; Bornatowski, Robert & Costa, 2007). However, Bornatowski et al. (2011) reported an unusual and targeted fishing effort using large mesh (>40 cm) gillnets baited with rays or bony fishes to capture larger sharks, resulting in landings in which juvenile tiger sharks made up 17.6% of all catches. That indicates a yet uninvestigated local market. According to the authors, targeted efforts may be due to larger sharks being commonly sold directly to specific middlemen and fish markets, attaining a relatively high shark meat value, of up to $3.00/kg USD, while fins reach up to $70.00/kg USD, a significant income for artisanal fishers. Another study retrieved in our review reports that tiger sharks are exposed to higher commercial fishing pressure in Northeastern Brazil as at least 23% of previously monitored animals caught in a post-capture survival study were incidentally captured by coastal or oceanic fisheries in less than 2 months following release (Afonso & Hazin, 2014). According to the authors, switching traditional hooks for circular hooks has been suggested as an alternative to reduce the mortality of non-targeted captured sharks, including tiger sharks. However, while reducing mortality, the use of circle hooks increased tiger shark capturs in both pelagic and bottom longlines in Northeastern Brazil (Afonso et al., 2011).

The constant incidental capture of neonates and juveniles and the increase of commercial fishing targeting sharks in the South Atlantic raises concerns about the conservation status of the species. In addition, the lack of adequate commercial fisheries data (FAO, 2020), and the fact that many countries group different shark and ray species into the generic category of “shark”, “cação” or “cazón”, demonstrates an additional challenge to fisheries management and species conservation. More importantly, the lack of data for all other South Atlantic regions, especially the Western portion, is of extreme concern and should be addressed as a priority in the next few years. Studies on regionalized fishing profiles (*e.g*., artisanal, industrial, fishing gear categories) are paramount. Furthermore, the assessment of regionalized fish stocks, identification of which fishing fleets and gear are responsible for the greatest pressures are also required, all leading to regionalized management plans for the species.

#### Attacks on humans

As tiger sharks exhibit coastal and generalist habits, their distribution areas routinely overlap with environments commonly used by humans, increasing the chances of negative interactions (International Shark Attack Files, 2022). On a global level, this species ranks second concerning unprovoked attacks on humans, only after the Great White Shark (*Carcharodon carcharias*) (International Shark Attack Files, 2022), although depending on the area (*e.g*., Recife, Hawaii, and South Africa), they are considered the main species involved in the attacks.

Most recovered studies (*n* = 6) concerning attacks on human focus on the Brazilian coast, associated with a high number of unprovoked shark attacks in general in the state of Recife, with 47 recorded cases from 1992 to 2006. The responsible species were, however, only identified in seven cases (14.9%), with a single case attributed to a tiger shark specimen (Hazin, Burgess & Carvalho, 2008), performed by Gadig & Sazima (2003) based on a tooth fragment found in the victim’s lesion. The overall size of the identified sharks involved in the seven attack cases was estimated to be between 1 and 3 m TL, and at least one shark was described after a non-fatal encounter as being “small”, potentially indicating coastal preferences for juveniles and sub-adults, as in other species (Hazin, Burgess & Carvalho, 2008).

The significant increase in attacks in Recife is postulated by the authors as caused by anthropogenic activities, such as increased vessel traffic due to the Suape Port construction in the state of Pernambuco and increased dumping of organic matter from slaughterhouses and vessels (Hazin, Burgess & Carvalho, 2008). This hypothesis was later partially supported by Hazin et al. (2013), who obtained telemetry data from five tagged tiger sharks captured off the coast of Suape, all of which were juveniles (<2 m TL). However, a higher number of tagged animals from different size classes is required to prove this hypothesis. Furthermore, tiger shark movements on a larger temporal scale (seasonality) should also be further investigated, as well as their origins, whether they arrive from the North Atlantic (*e.g*., Florida coast, Gulf of Mexico, Bahamas), the Eastern Atlantic (Oceanic domain or African coast) or Southern Atlantic (Southeast and Southern Brazil) waters bordering the Brazilian coast, to aid in the development of effective shark attack mitigation measures.

In response to the expressive number of lethal attacks in Recife, the Recife Shark Monitoring Program (PMTR—acronym in Portuguese) was created, comprising a non-lethal approach employing longlines and drumlines (Niella et al., 2021). The captured sharks were relocated to waters further from the coast instead of being slaughtered. The tiger shar was the most captured (73%) among potentially aggressive sharks, followed by the bull shark, *Carcharhinus leucas* (Afonso & Hazin, 2014). During PMTR operations, the number of attacks decreased by 97% compared to the previous period (Afonso & Hazin, 2014). In addition, during PMTR interruptions, the number of attacks increased to the same as prior to the program’s implementation. Another study evaluated the relationship between the seasonal coastal abundance of potentially aggressive sharks during PMTR operations, reporting that concomitant captures of both tiger and bull sharks were the most correlated with recorded bite records (Afonso, Garla & Hazin, 2017). Furthermore, the largest recorded animal (4.26 m) among all sharks was a tiger shark, supporting the hypothesis that a greater abundance of sharks leads to increased attacks, especially for potentially aggressive sharks.

It is important to note that the shark attacks in Recife exhibit high medical relevance, as they may lead to serious consequences for survivors, including microbial infections. In this context, Interaminense et al. (2010) investigated the presence of pathogenic bacteria in the oral cavities of tiger sharks and in the wounds of attack victims in Recife also testing their susceptibility to antibiotics. At least two pathogenic species, *Pseudomonas aeruginosa*, and *E. coli*, were found both in the sharks’ mouths and in the victims’ wounds, both being treatable with lesser spectrum antibiotics. Investigations of this nature are paramount to assist the Brazilian public health system in decision-making during shark attack victim treatment. However, despite the clear relevance of tiger sharks concerning human interactions, few studies for the South Atlantic are available. The lack of data on attacks for most countries bathed by the South Atlantic coast may be, in part, due to no implementation of attack recording systems whatsoever. For example, of all the countries bathed by the Atlantic Ocean where potentially dangerous sharks (white, tiger, and bull sharks) may occur, only the United States, Brazil, and South Africa have implemented some sort of attacking recording system and protective programs (McPhee, 2014). The majority of the African Continent, for example, lacks any type of records and relies mostly on media coverage (Trape, 2008). So, it is fair to assume that the number of negative interactions could be higher, as media coverage is limited (Trape, 2008).

Suggestions for further analysis on this topic include increasing the number of monitored animals and including individuals larger than 2 m TL, the investigation of possible differences in the potential for negative interactions according to sex and length, and species/length class identification through bite size. Furthermore, we also suggest investigations on from which directions the tiger sharks originate in areas presenting frequent attacks, especially Brazil, from the east (ocean waters), through the South Equatorial Current, or through the South, following the Brazilian coastline. The assessments of seasonal movements are also paramount, as are testing the influence of marine traffic and the dumping of organic matter as factors increasing tiger shark approaches. Finally, spatial ecology studies and the characterization of critical areas (*e.g*., nurseries) in regions where construction of large ports are planned are also imperative, aiming to establish control data in the event of attacks occurring after the establishment of these enterprises.

### Movements and migration

Of all reported tiger shark catches, 84.6% took place outside the original species distribution proposed (*e.g*., International Union for the Conservation of Nature, 2018; Ebert et al., 2013) (Domingo et al., 2016). Thus, a new distribution for the species based on data of pelagic longline vessels from several countries, such as the United States, Spain, Japan, Portugal, and Uruguay, was proposed (Domingo et al., 2016). Concerning the South Atlantic, all tiger shark individuals were captured beyond the range of their former distribution, also indicating distribution in waters off the continental shelf (Domingo et al., 2016). Furthermore, the expressive number of captures observed in equatorial waters between South America and Africa indicates a potential ecological corridor, highlighting the importance of collaborations between researchers from both regions to ensure adequate tiger shark’s management within the South Atlantic. In fact, tiger sharks captured and tagged at the Fernando de Noronha Archipelago have been shown to move to distant offshore areas and reach different Southwest African areas (Afonso, Garla & Hazin, 2017). These data suggest the Archipelago as a connecting site for different occurrence areas, linking Atlantic and Indo-Pacific Oceans populations, with mounting evidence from different research fields (Bernard et al., 2016; Domingo et al., 2016; Afonso, Garla & Hazin, 2017; Carmo et al., 2019). In addition, juvenile tiger sharks that inhabit coastal Northeastern Brazil areas seem to use the region as a disperser point for the pelagic environment (Afonso, Garla & Hazin, 2017).

Furthermore, tiger sharks seem to alter their habitats as they grow, expanding their habitats towards the oceanic environment (Afonso & Hazin, 2015), suggesting distribution per ontogenetic stage and movement driver differences (Afonso, Garla & Hazin, 2017). This has been confirmed in the West Atlantic, which also comprises differences in distribution by sex, with a greater number of juveniles of both sexes, measuring <200 cm in TL inhabiting shallow coastal waters (Afonso & Hazin, 2014; Afonso, Garla & Hazin, 2017; Domingo et al., 2016). The occurrence of neonates has also been reported for shallower neritic waters, while a predominance of immature females between 200–260 cm in TL is noted in areas far from the coast (Afonso, Garla & Hazin, 2017). Regarding vertical distribution, juveniles are more predominant between surface waters (>5 m) up to 60 m in depths, although some reports indicate incursions up to 1,000 m (Afonso & Hazin, 2015). As tiger sharks grow from 150 to 300 cm in TL, their maximum diving depth increases up to four-fold, allowing the species to explore different environments, decreasing intraspecific competition, and expanding their ecological niche (Afonso & Hazin, 2015).

In one of the few studies carried out exclusively outside Brazil, tiger sharks tagged around Ascension Island, a British isolated volcanic island located 7°56′ south of the Equator, moved from shallow waters through the thermocline (where the temperature drops from 13.5 to 6 °C) (Madigan et al., 2021). Most dives took place at >20 °C, although frequent dives to lower temperatures were noted, albeit limited to the thermocline floor (150 m). Dissolved oxygen (DO) during dives ranged from 2 to 5 mL^−1^, with animals expending a considerable time (8.3%) at 2 mL^−1^, suggesting hypoxia tolerance in the species (Madigan et al., 2021). However, as only one tiger shark was monitored, caution is advised in interpreting the presented data.

Except for Recife and Fernando de Noronha, all occurrence tiger shark data found and included herein are from commercial fishing fleets. Thus, targeted capture assessments or more comprehensive monitoring of commercial fleets are required to further increase knowledge on the species’ area of occurrence. Furthermore, telemetry assisted studies would also be a valuable tool to obtain further information on tiger shark movements and migration, although costs must be considered, as cited in the population structure topic. Evaluations on movements along the Brazilian coast, and the southwest-south connection of Brazil, and the Northeastern regions are mandatory for this topic. It would also be interesting to evaluate if juveniles from other regions carry out the same oceanic incursions. Furthermore, it is paramount to understand if the connection between West and East Atlantic is carried out only by Fernando de Noronha or if it also takes place in the Southeast-South regions of Brazil, to identify the connectivity between sharks in the West and East Atlantic through migratory movements.

### Population genetics

Although tiger sharks exhibit a circumglobal distribution in both tropical and temperate waters, information on population connectivity, dynamics and genetic structure, only began to be unraveled in the last decade (Andrade et al., 2021; Bernard et al., 2016; Carmo et al., 2019). The studies retrieved herein indicate confirmed genetic differentiation between tiger sharks from the West Atlantic, North Atlantic, and Southwest Atlantic, and an isolated population identified in Hawaii (Andrade et al., 2021; Bernard et al., 2016). Studies indicate that the separation between Indo-Pacific and West Atlantic populations took place only about 1 million years ago, with the Southwest Atlantic serving as a connection point between ocean basins, allowing for population breeding through incursions along the South African tip (Bernard et al., 2016). Furthermore, mitochondrial DNA analyses indicate a matrilineal intra-oceanic population structure, where the gene flow is highly influenced by females, mainly by philopatry (Bernard et al., 2016). It is important to note that this may lead to different fishing vulnerabilities between males and females, in turn resulting in genetic diversity imbalances in the long term, due to altered male to female ratios and pregnant female densities and, consequent significant diversity losses. Thus, efforts to conserve the species should be revised considering these differences (Bernard et al., 2016).

Atlantic and Pacific population differences were confirmed in further studies by Carmo et al. (2019), who also obtained new relevant information on the local barriers that affect tiger shark gene flows. This author reported three highly structured populations in the Atlantic, off the coasts of the United States, Panama, and Brazil, with two distinct populations centered in the Brazilian states of Pará, in northern Brazil, and São Paulo, in southeastern Brazil. The Fernando de Noronha Archipelago population exhibits a genetic structure with haplotypes from all Atlantic populations, comprising the most diverse population (Andrade et al., 2021; Carmo et al., 2019), denoting breeding between tiger sharks from both hemispheres, connecting the North Atlantic to the South Atlantic, with the Southwestern Atlantic in fact comprising a bridge between populations, as noted previously by other authors (Bernard et al., 2016). Thus, Fernando de Noronha has again been noted as a tiger shark convergence center, reinforcing its ecological and conservation relevance (Andrade et al., 2021; Carmo et al., 2019).

Decreases in the genetic variability of tiger sharks from the South Atlantic have been described by Mendes et al. (2016), excluding Fernando de Noronha individuals. Interestingly, the Florida population (Gulf of Mexico), one of the most studied globally, presents only 1.7% of the observed diversity of the Fernando de Noronha Archipelago (Carmo et al., 2019). From a conservationist point of view, these data reveal significant tiger shark fragility and vulnerability at regional levels. Isolated populations are also more likely to lose genetic diversity through overfishing, as gene flow through migration events is very low (Carmo et al., 2019). Therefore, the Fernando de Noronha Archipelago should be recognized as a tiger shark sanctuary, due to its essential gene flow role and consequent maintenance of healthy populations (Andrade et al., 2021; Carmo et al., 2019).

Genetic tools are paramount in unveiling population structure, through parameters such as genetic drift, mutation, and selection (Holmes et al., 2017) and genetic connectivity dynamics, also allowing for historical samples to be genotyped and assigned back to their population of origin (Sort et al., 2021). In fact, knowledge in this regard for many large-bodied, highly migratory, apex predator sharks across their global ranges are still very limited (Bernard et al., 2016). Thus, although a relatively high amount of tiger shark population genetics data is reported in the retrieved studies in our review, it is clear that further assessments are required, although genetic techniques, similarly to telemetry methods, are also relatively prohibitive in certain regions, due to high costs. Some efforts to create and validate quick, low cost and easily applicable methods, such as PCR analysis based on 5S rDNA repeat unit amplification have been noted, to contribute to conservation efforts in general (Natesh et al., 2019) and even specifically to shark conservation management (Pinhal et al., 2008), although these are relatively scarce and spaced between. Increasing sampling events is required, also focusing on islands and oceanic outcrops to identify potential new points of convergence. The number of animals sampled in the West and mainly, the East Atlantic, should be increased, aiming to investigate the presence of regional subpopulations. The comparison of populations more susceptible to human pressures (mainly fisheries) with more protected populations also allows for assessments on the potential loss of tiger shark genetic diversity.

In short, many knowledge gaps were noted, even for more traditional studies. The situation worsens when new methodologies are considered, as the associated costs are a prohibitive factor. Table 1 below presents knowledge gaps and questions not yet investigated or that require more effort in view of each of the topics discussed herein.

**Table 1:** Tiger shark knowledge gaps and questions not yet investigated or that require more effort in view of each of the topics discussed in this review.

Research area	Topics covered	Knowledge gaps/Future directions	Tools and methods suggested
Morphology and systematics	Dermal denticles and caudal fin shape	Morphological variations such as size, shape, and allometry (external, internal and dental) associated with ontogeny, sexual dimorphism and between populations	Linear morphometry and geometric morphometry; Investigations addressing ecomorphological and phylogenetic aspects; Use of material deposited in zoological collections; Use of photographic records with scale (citizen science, social networks and open-access databases); Monitoring commercial fishing landings to obtain samples; Interviews and visits to fishing communities that have preserved materials such as jaws to obtain morphological data
Reproduction	Spermatozoa in the nidimentary glands; Estimated minimum size for sexual maturation; Estimated litter size and parturition periods; Adult male sexual traits	Characterization of the reproductive cycle and fecundity; Confirmation of minimum sexual maturation sizes, parturition periods/areas, and litter size; Validation of non-lethal methods to access reproductive traits	Monitoring commercial fishing landings for collection of reproductive organs and offspring; description of anatomy and histology of reproductive organs; access to traditional knowledge to map possible parturition and nursery areas; validation of ultrasound and hormonal analysis to assess fertility and confirm pregnancy
Age and growth	Mark-and-recapture data	Validate age and growth through vertebrae analysis (top priority); Additional mark-and-recapture studies	Collaboration with commercial fishers to collect vertebrae; Collaboration with recreational fishers for mark-and-recapture studies
Feeding ecology	Diet and trophic position	Advance knowledge of the diet and trophic interactions of the species in other regions; Validation of non-lethal methods	Partnership with commercial fishers to collect samples; Stable isotopes in muscle samples and regurgitation validation for stomach content
Physiology	Stress upon scientific capture; Disease; Steroid hormones x migration	Capture stress from commercial fisheries, along with survival rates; Validation of proxies to predict on-vessel mortality; Health monitoring of wild individuals; Physiological screening across life stages and sexes	Partnership with commercial fishers to collect samples; Biochemical and molecular analysis
Population structure	Coastal abundance and correlation to tide; Sex ratio; Ontogenetic abundance and seasonality.	Expansion of studies to poor-data regions in both 41 and 47 fishing areas	Partnership with commercial and recreational fishers and access to traditional knowledge
Human interactions	Attacks Fisheries	Resume shark attack control programs and monitoring; Improve methods to identify species involved in the attacks; Improve data on shark attack medicine Improve commercial fisheries data; Investigate artisanal fisheries’ impacts on the species; Generate data for other regions, especially on the 47-fishing area; Track tiger shark marketing and productive chains	Non-lethal captures and release far from the coast; Linear and Geometric morphometry of teeth, arches, and, when possible, of wounds; Creation of a specialized service center for attacks on the public health system; First responder training; Remote electronic monitoring in industrial fleets along with monitoring of landings and fishing markets, artisanal landings; Co-participatory management with fishers; Access to traditional knowledge
Movements and migration	Shore to ocean movements; Transoceanic movements; Ontogenetic variation on movements; Vertical movements and correlation to abiotic factors	Intrinsic and extrinsic drives of movement and migration; Expansion of studies to poor-data regions in both 41 and 47 fishing areas	Physiological and ecological analysis; Telemetry; Data from stranded sharks; Partnership with commercial and recreational fishers and access to traditional knowledge
Distribution	Range expansion in the Atlantic Basin; Coastal distribution; Ontogenetic distribution	Sex segregation; Parturition and nursery areas; Expansion of studies to poor-data regions in both 41 and 47 fishing areas	Telemetry; Data from stranded sharks; Partnership with commercial and recreational fishers and access to traditional knowledge
Population genetics	Connections and differentiation between the oceanic populations and between the Atlantic subpopulations	Philopatry; Multiple Paternity; Genetic diversity driven by fisheries	Partnership with fishers to collect samples; Multidisciplinary approach (morphometric, reproductive and genetic data)

As some significant knowledge gaps on major research areas were also observed, a summary on future directions is also presented (Table 2).

**Table 2:** Future tiger shark study directions.

Research area	Future directions
Ecotoxicology	Screening of traditional and emerging contaminants in different tissues, life stages, and sexes; Meat consumption risks; Assessment of the lethal and sub-lethal effects of pollution at an individual and population level
Climate change	Effects of climate change on metabolic and respiratory scopes, and on reproductive processes; Thermal tolerance limits and distribution patterns; Possible lethal and sub-lethal effects of rising temperatures and ocean acidification
Microbiology	Associated microbiota and possible harmful effects on animal health; Oral microbiota associated with the severity of bite wounds
Parasitology	Taxonomic description of endo and ectoparasites associated with the species and possible deleterious effects on animal health; Parasite x host co-evolution studies
Conservation	Proposition of fisheries and spatial management measures for the species, considering the human dimensions intrinsic to exploitation
Public policies	Proposition of legislation based on minimum and maximum capture sizes, quotas and seasonal/spatial closures
Marine protected areas	Delineation of habitat use and critical areas for MPAs proposition; Evaluation of the effectiveness of existing MPAs for the species
Ecological fisher knowledge	Studies to characterize artisanal fisheries production, biology, and ecology of the species; Recognition of traditional knowledge as a source of valuable information in data-poor regions

## Conclusions

From the data compiled in the present study, it is clear that tiger sharks receive little or no attention in the South Atlantic. This is very interesting considering how much attention and priority the species receives in other regions, in addition to being constantly featured in TV shows and documentaries. In fact, with the exception of data from the states of Pernambuco and Paraná (Northeast and Southern Brazil, respectively), nothing is known about tiger sharks in the western South Atlantic. The situation is even worse for the eastern South Atlantic, as only one study, in which the tiger shark was not the focus, has been published so far. This is particularly worrying considering how important subsistence fisheries are in these regions, in addition to the major challenges for commercial fisheries’ adequate management. Thus, priority should be given to studies on other regions, especially in African waters.

Some possible reasons for the lack of data are noted. First, it is possible that the low number of individuals landed is a limiting factor for studies with tiger sharks in these regions, as captures are predominantly incidental. Second, the lack of financial investment, a recurring problem in the countries bordering the South Atlantic, can also be a very limiting factor. It is important to mention that challenges related to parachute or colonizing science should also be recognized and properly addressed. This is due to the fact that, as the species has great appeal in other countries, recognizing the severe knowledge gaps in this region can open space for researchers from other nations to empower themselves in investigations. Thus, it is necessary that the government of the countries bordering the South Atlantic, along with national and international funding agencies, recognize the importance of financing local projects. One way to strengthen research groups in these regions is through the creation of a coalition, aiming to join efforts to generate the necessary data. Furthermore, it is of paramount importance that more institutions and non-governmental organization take responsibility for generating the necessary data as, so far, little or none of the available data is sufficient to assess the local risk of extinction of the species or propose more comprehensive management plans.

## Supplemental Information

10.7717/peerj.14750/supp-1 Supplemental Information 1 Results of the scientometric search strategy on Tiger Shark literature for South Atlantic waters in chronological sequence.

## Additional Information and Declarations

### Data Availability

The following information was supplied regarding data availability: The paper is a literature review and does not contain raw data.
