# Supplementary material for: Almost nothing is known about the tiger shark in South Atlantic waters
Source: PeerJ. 2023 Jan 20;11:e14750. doi: 10.7717/peerj.14750 (PMC9869778; doi:10.7717/peerj.14750)
Supplement: Supplemental Information 1 [file peerj-11-14750-s001.docx]

**Table S1.** Results of the scientometric search strategy on Tiger Shark literature for South Atlantic waters in chronological sequence.

| **Authors(s)** | **Date** | **Topics** | **Major Fishing Areas (FAO)** | **Region/Country** |
| --- | --- | --- | --- | --- |
| Afonso et al. | 2012 | Age and growth | Atlantic, Southwest | Northeast/Brazil |
| Brandão et al. | 2016 | Biological revision | Atlantic, Pacific and Indic | Several (Atlantic, Pacific, and Indic) |
| Domingo et al. | 2016 | Distribution | Atlantic | Several (Northwest/Britain; Northeast and Southeast/US; Central Atlantic; Northeast and Southeast/Brazil; Northwest and Southwest/ Africa) |
| Hazin et al. | 2017 | Distribution | Atlantic, Southwest | Northeast/Brazil |
| Lopes et al. | 2020 | Distribution | Atlantic, Southwest | Southeast/Brazil |
| Rosas et al. | 1992 | Feeding ecology | Atlantic, Southwest | Southeast/Brazil |
| Di Beneditto | 2004 | Feeding ecology | Atlantic, Southwest | Southeast/Brazil |
| Bornatowski et al. | 2007 | Feeding ecology | Atlantic, Southwest | Southern/Brazil |
| Barbosa-Filho et al. | 2014 | Feeding ecology | Atlantic, Southwest | Northeast/Brazil |
| Bornatowski et al. | 2014 | Feeding ecology | Atlantic, Southwest | Southern/Brazil |
| Bornatowski et al. | 2014 | Feeding ecology | Atlantic, Southwest | Southern/Brazil |
| Rada et al. | 2015 | Feeding ecology | Atlantic, Southwest | Northeast/Brazil |
| Barbosa-Filho & Costa-Neto | 2016 | Feeding ecology | Atlantic, Southwest | Northeast/Brazil |
| Shibuya et al. | 2017 | Feeding ecology | Atlantic, Southwest | Northeast/Brazil |
| Miranda et al. | 2021 | Feeding ecology | Atlantic, Southwest | Southeast/Brazil |
| Gadig & Sazima | 2003 | Human interactions | Atlantic, Southwest | Northeast/Brazil |
| Costa & Chaves | 2006 | Human interactions | Atlantic, Southwest | Southern/Brazil |
| Hazin et al. | 2008 | Human interactions | Atlantic, Southwest | Northeast/Brazil |
| Iteraminense, et al. | 2010 | Human interactions | Atlantic, Southwest | Northeast/Brazil |
| Afonso et al. | 2011 | Human interactions | Atlantic, Southwest | Northeast/Brazil |
| Bornatowski et al. | 2011 | Human interactions | Atlantic, Southwest | Southern/Brazil |
| Hazin & Afonso | 2014 | Human interactions | Atlantic, Southwest | Northeast/Brazil |
| Poscai et al. | 2017 | Morphology and systematics | Atlantic, Southwest | Southeast/Brazil |
| de Oliveira et al. | 2019 | Morphology and systematics | Atlantic, Southwest | Southeast/Brazil |
| Hazin et al. | 2013 | Movements and migration | Atlantic, Southwest | Northeast/Brazil |
| Afonso & Hazin | 2014 | Movements and migration and Human interactions | Atlantic, Southwest | Northeast/Brazil |
| Afonso & Hazin | 2015 | Movements and migration | Atlantic, Southwest | Northeast/Brazil |
| Afonso et al. | 2017 | Movements and migration | Atlantic, Southwest | Northeast/Brazil |
| Afonso et al. | 2017 | Movements and migration and Human interactions | Atlantic, Southwest | Northeast/Brazil |
| Madigan et al. | 2020 | Movements and migration | Eastern Atlantic | Southwest/England |
| Wosnick et al. | 2018 | Physiology and Movements and migration | Atlantic, Southwest | Northeast/Brazil |
| Wosnick et al. | 2017 | Physiology | Atlantic, Southwest | Northeast/Brazil |
| Wosnick et al. | 2020 | Physiology | Atlantic, Southwest | Southern, Southeast and Northeast/Brazil |
| Bernard et al. | 2016 | Population Genetics | Atlantic, Pacific and Indic | Several (Southeast/US; Southern/ Brazil; western South Indian Ocean/South Africa; Adaman Sea/Indonesia; eastern and western south Pacific/Australia; Central Pacific/Hawaii) |
| Mendes et al. | 2016 | Population Genetics | Atlantic, Southwest | Southeast and Northeast/Brazil |
| Carmo et al. | 2019 | Population Genetics | Atlantic | Several (Northeast and Southeast/US; Central Atlantic/Panama; Northeast and Southeast/Brazil; Pacific) |
| Andrade et al. | 2021 | Population Genetics | Atlantic, Southwest | Several (Southeast/US; Northeast and Southern/ Brazil; Southwestern Indian Ocean/South Africa and Reunion Island; Adaman Sea/Indonesia; Southeastern and Southwestern Pacific/Australia; Central Pacific/Hawaii) |
| Afonso et al. | 2014 | Population structure | Atlantic, Southwest | Northeast/Brazil |
| Alves | 1977 | Reproduction | Atlantic, Southwest | Northeast/Brazil |
| Bornatowski et al. | 2012a | Trophic ecology | Atlantic, Southwest | Northeast/Brazil |
| Bornatowski et al. | 2012b | Trophic ecology | Atlantic, Southwest | Northeast/Brazil |
|  |  |  |  |  |
